# Inclusion of Health Equity Initiatives in Hospitals' Strategic Plans

**DOI:** 10.1089/heq.2023.0183

**Published:** 2023-11-27

**Authors:** Simone R. Singh, Cherie Conley

**Affiliations:** ^1^ Department of Health Management and Policy, University of Michigan School of Public Health, Ann Arbor, Michigan, USA.; ^2^ University of Michigan School of Nursing, Ann Arbor, Michigan, USA.

**Keywords:** health equity, social determinants of health, hospitals, strategic planning, accountability

## Abstract

**Objective:**

This study examined the health equity initiatives included in US hospitals' strategic plans.

**Methods:**

Using data from the American Hospital Association (AHA) 2021 Annual Survey, the study described the types of health equity initiatives that US hospitals included in their strategic plans. The analysis focused on the following seven initiatives: (1) equitable and inclusive organizational policies; (2) systematic and shared accountability for health equity; (3) diverse representation in hospital and health care system leadership; (4) diverse representation in hospital and health care system governance; (5) community engagement; (6) collection and use of segmented data to drive action; and (7) culturally appropriate patient care. Logit and zero-truncated Poisson regression analysis was used to examine organizational and community-level characteristics of hospitals with the most comprehensive health equity strategic plans.

**Results:**

Of the 4359 general medical and surgical hospitals that completed the AHA's 2021 survey, 45.1% provided complete information on their health equity strategies. The comprehensiveness of hospitals' health equity efforts varied across organizations. Regression analysis showed that larger hospitals, nonprofit hospitals, and hospitals affiliated with health systems tended to have more comprehensive health equity initiatives as did hospitals located in urban areas, hospitals in communities with higher household incomes, and hospitals in communities with greater proportions of Hispanic residents.

**Conclusions:**

While improving health and health equity is a key aspect of many hospitals' missions, the extent to which hospitals include health equity initiatives into their strategic plans varied noticeably. Committing to a comprehensive set of efforts aimed at improving health equity requires human and financial resources as well as dedicated leadership.

## Introduction

The COVID-19 pandemic has brought to light significant disparities in population health outcomes and the insufficient progress that has been made toward reducing health disparities.^1,2^ In this challenging environment, hospitals have emerged as crucial players in addressing the root causes of health inequities.^3,4^ As key community members, hospitals possess the necessary resources, expertise, and influence to invest in the social, economic, and environmental determinants of health and promote health equity.

Traditionally, hospitals have focused on improving access to high-quality clinical care for marginalized and disadvantaged groups as part of their efforts to promote health equity.^5^ This approach, aimed at improving health care delivery and addressing health disparities, encompasses a range of initiatives implemented within hospitals.

One essential component of this approach involves utilizing data to identify and address disparities in health care access, treatment, and outcomes among different populations. Another crucial aspect of this approach is enhancing language and cultural competency within the hospital setting by investing in training programs and resources to ensure that health care providers can effectively communicate and understand the needs of patients from different cultural and linguistic backgrounds.

Furthermore, hospitals prioritize diversity, equity, and inclusion initiatives to build a more diverse workforce, ensure equity among staff members and patients, and cultivate working environments in which all employees feel included—all with an ultimate goal of achieving health equity.^6^

Nevertheless, to effectively tackle the social, economic, and environmental factors that impact health, hospitals must allocate resources to endeavors that extend beyond their physical boundaries.^7^ These efforts encompass investments in both macrolevel (upstream) and microlevel (downstream) social determinants of health.^8^ To this end, hospitals actively participate in community outreach and establish collaborative relationships with public health agencies and local community-based organizations.

By partnering with other community members, hospitals can identify and comprehend issues, devise and execute solutions, and gauge their effectiveness.^9^ Moreover, hospitals engage in health policy advocacy and lend support to initiatives that seek to bring about systemic changes fostering health equity.

This study provides insights into the specific efforts that hospitals engage in to promote health equity. Using data from the 2021 American Hospital Association (AHA) Annual Survey, the study answers the following research questions: (1) What types of health equity initiatives do hospitals commonly engage in? (2) How do hospitals with more comprehensive health equity initiatives differ from hospitals with less comprehensive health equity initiatives?

Study findings can provide health practitioners and policymakers with insights into the prevalence of health equity initiatives among hospitals in the United States. Findings also highlight key characteristics of hospitals with more comprehensive health equity initiatives, thus informing practitioners and policymakers about the factors that facilitate or impede hospital attempts to engage in efforts aimed at improving health equity.

## Materials and Methods

### Data and sample

Data for this study came from AHA's 2021 Annual Survey. The 2021 Annual Survey included several questions related to hospitals' health equity initiatives, which were the focus of this study. In 2021, 4359 nonfederal general medical and surgical hospitals completed the annual survey. Of these, 1964 hospitals (45.1%) provided complete data on their health equity initiatives. The remaining 2395 hospitals (54.9%) did not complete the survey questions on their health equity initiatives and were excluded from the analysis.

Additional data on community-level characteristics were obtained from the Area Health Resources Files (AHRF) compiled and published by the Health Resources and Services Administration (HRSA). Since no human subjects were involved in this research, IRB approval was not obtained for this work.

Hospitals that provided complete data on their health equity initiatives differed notably from those that did not complete these survey questions (Table 1). Hospitals with complete data tended to be larger organizations, nonprofit organizations, and organizations affiliated with health systems. These hospitals tended be located in urban areas and were less likely to have a critical access hospital designation. These hospitals also tended to be in communities with somewhat higher household incomes and lower uninsured rates. Of note, these hospitals with complete data also served somewhat more racially and ethnically diverse communities.

**Table 1.** Organizational and Community-Level Characteristics of Hospitals With and Without Complete Data on Health Equity Initiatives, American Hospital Association Annual Survey 2021

Characteristic	Hospitals with complete data on health equity initiatives (***n***=1964)	Hospitals without complete data on health equity initiatives (***n***=2395)	***t***-Test of differences in means, ***p***-value
Organizational characteristics			
Bed size, mean (SD)	224 (257)	121 (151)	<0.01
System affiliation, *n* (%)	1547 (78.8)	1349 (56.3)	<0.01
Ownership, *n* (%)			<0.01
Nonprofit	1552 (79.0)	1223 (51.1)	
For-profit	138 (7.0)	501 (20.9)	
Government	274 (14.0)	671 (28.0)	
Critical access hospital, *n* (%)	398 (20.3)	949 (39.6)	<0.01
Community-level characteristics			
Median household income, mean (SD)	$64,572 ($17,451)	$58,776 ($15,973)	<0.01
Uninsured rate for those younger than 65 years, mean (SD)	10.7% (5.0%)	11.7% (5.0%)	<0.01
Percentage of residents who are Black, mean (SD)	10.6% (12.6%)	9.8% (13.9%)	<0.05
Percentage of residents who are Hispanic, mean (SD)	14.8% (15.8%)	13.0% (15.7%)	<0.01
Urban–rural location			<0.01
Urban location, *n* (%)	1341 (68.3)	1131 (47.2)	
Rural location, *n* (%)	623 (31.7)	1264 (52.8)	

SD, standard deviation.

### Outcome measures

The main variables of interest for this study were the types of health equity initiatives that hospitals reported to have included in their strategic plans. Data for these variables came from hospitals' responses to the following AHA Annual Survey question: “Does your hospital or health care system have a health equity strategic plan for the following?”

Seven answer choices were available to respondents: (1) equitable and inclusive organizational policies; (2) systematic and shared accountability for health equity; (3) diverse representation in hospital and health care system leadership; (4) diverse representation in hospital and health care system governance; (5) community engagement; (6) collection and use of segmented data to drive action; and (7) culturally appropriate patient care.

Each answer choice was coded as a binary variable whereby a value of one indicated that the hospital had a strategic plan for the respective type of health equity initiative. In addition to examining the seven binary variables, a count variable was created to assess the comprehensiveness of a hospital's health equity strategic plan by summing up the seven binary variables.

### Organizational characteristics

All regression analyses included a key set of organizational characteristics. We used the number of staffed beds, as reported by hospitals in the AHA Annual Survey, as a proxy for a hospital's size. Ownership was defined as nonprofit, for-profit, or government owned. System affiliation was defined as membership in a health system, as reported in the AHA Annual Survey. Finally, critical access hospitals included all hospitals with a critical access hospital designation by the Centers for Medicare and Medicaid Services (CMS).

### Community characteristics

Because hospitals operate in the context of their communities, we included a set of key community-level indicators into the analysis, including median household income of the county a hospital is located in; the county-level uninsured rate measured as the percentage of residents younger than age 65 without health insurance coverage; and racial composition of the population in a county measured as the percentages of county residents who are Black and Hispanic.

Urban–rural location was defined using urban–rural continuum codes by the US Department of Agriculture, whereby metro counties were classified as urban locations and nonmetro counties were classified as rural locations.

### Analytical strategy

Descriptive analysis was used to describe the types of health equity initiatives included in hospitals' strategic plans. Organizational characteristics of hospitals associated with each of the seven binary variables described above were examined using logit regression analysis. To explore organizational characteristics of hospitals associated with the comprehensiveness of health equity strategic plans, we conducted zero-truncated Poisson regression analysis.

Zero-truncated Poisson regression analysis can be used to model count data for which the value zero cannot occur, as is the case for the indicator of comprehensiveness of health equity strategic plans examined in this study. All hospitals that completed the section on health equity in the 2021 AHA Annual Survey reported including at least one of seven possible health equity initiatives in their strategic plans.

To ensure that overdiversion was not a problem and a Poisson model was appropriate for modeling data, we computed the ratio of the Pearson chi-square statistic to its degrees of freedom (0.79) and verified that it was less than one. We computed robust standard errors for all regression coefficients.

## Results

### Descriptive results

**FIG. 1.**
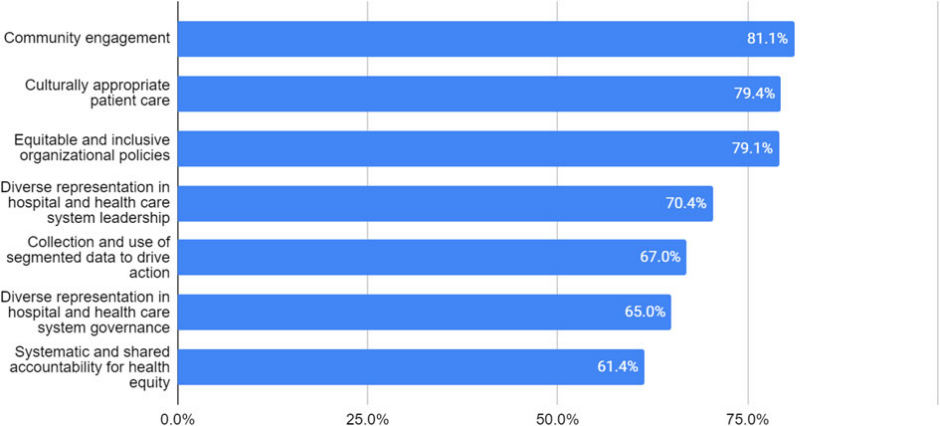
Health equity initiatives included in hospitals' strategic plans, 2021. Note: *n*=1964 hospitals.

The most common type of health equity initiative included in hospitals' strategic plans was “community engagement” (81.1%), followed by “culturally appropriate patient care” (79.4%) and “equitable and inclusive organizational policies” (79.1%) (Fig. 1). Somewhat less common initiatives included “diverse representation in hospital and health care system leadership” (70.4%), “collection and use of segmented data to drive action” (67.0%), and “diverse representation in hospital and health care system governance” (65.0%).

The least commonly included initiative was “systematic and shared accountability for health equity” (61.4%). On average, hospitals reported 5.1 health equity initiatives with a median of 6 and an interquartile range of 3–7 initiatives. Around 40% of sample hospitals reported including all seven health equity initiatives in their organization's strategic plan.

### Multivariate results

Logit regression analysis showed that system-affiliated hospitals had consistently higher odds of having implemented any of the seven types of health equity initiatives included in the AHA Annual Survey (Table 2). Nonprofit hospitals had higher odds of having implemented most of the seven types of health equity initiatives compared with for-profit and government-owned hospitals.

**Table 2.** Organizational and Community-Level Characteristics Associated with Key Components of Hospitals' Health Equity Strategic Plans

	Equitable and inclusive organizational policies	Systematic and shared accountability for health equity	Diverse representation in hospital and health care system leadership
Organizational characteristics			
Bed size	1.00 (0.00030)	1.00^*^ (0.00024)	1.00 (0.00026)
Ownership			
Nonprofit	Reference group	Reference group	Reference group
For-profit	0.55^**^ (0.13)	0.46^**^ (0.092)	1.03 (0.25)
Government	0.74 (0.12)	0.93 (0.14)	0.51 (0.079)
System member	2.61^**^ (0.36)	2.77^**^ (0.35)	2.23^**^ (0.29)
Critical access hospital	1.45^*^ (0.24)	1.11 (0.16)	1.41^*^ (0.21)
Community-level characteristics			
Median household income	1.00 (0.0000044)	1.00 (0.0000035)	1.00^**^ (0.0000041)
Uninsured rate for those younger than 65 years	0.99 (0.015)	0.98 (0.013)	1.03 (0.015)
% Black residents	0.98^**^ (0.015)	0.99 (0.0044)	1.01 (0.0050)
% Hispanic residents	1.00 (0.0048)	1.01^*^ (0.0040)	1.01^*^ (0.0045)
Urban–rural location			
Rural location	Reference group	Reference group	Reference group
Urban location	1.26 (0.21)	1.06 (0.15)	1.23 (0.19)

	Diverse representation in hospital and health care system governance	Community engagement	Collection and use of segmented data to drive action
Organizational characteristics			
Bed size	1.00 (0.00023)	1.00 (0.00030)	1.00 (0.00027)
Ownership			
Nonprofit	Reference group	Reference group	Reference group
For-profit	0.89 (0.19)	0.37^**^ (0.082)	0.29^**^ (0.059)
Government	0.74^*^ (0.11)	0.55^**^ (0.094)	0.57^**^ (0.089)
System member	2.70^**^ (0.34)	1.36^*^ (0.20)	2.50^**^ (0.32)
Critical access hospital	1.49^**^ (0.22)	0.95 (0.16)	1.29 (0.19)
Community-level characteristics			
Median household income	1.00 (0.0000037)	1.00^*^ (0.0000046)	1.00^**^ (0.0000040)
Uninsured rate for those younger than 65 years	0.99 (0.013)	1.03 (0.016)	0.97 (0.013)
% Black residents	1.00 (0.0046)	1.00 (0.0054)	1.01 (0.0049)
% Hispanic residents	1.01^*^ (0.0042)	1.00 (0.0048)	1.01^*^ (0.0043)
Urban–rural location			
Rural location	Reference group	Reference group	Reference group
Urban location	1.20 (0.17)	1.25 (0.21)	1.25 (0.11)

	Culturally appropriate patient care		
Organizational characteristics			
Bed size	1.00 (0.00031)		
Ownership			
Nonprofit	Reference group		
For-profit	0.69 (0.17)		
Government	0.66^*^ (0.11)		
System member	1.50^**^ (0.21)		
Critical access hospital	1.56^**^ (0.25)		
Community-level characteristics			
Median household income	1.00 (0.0000044)		
Uninsured rate for those younger than 65 years	1.02 (0.016)		
% Black residents	0.99 (0.0051)		
% Hispanic residents	1.01 (0.0050)		
Urban–rural location			
Rural location	Reference group		
Urban location	1.10 (0.18)		

Note: Table shows odds ratios from logit regression models with standard errors in parentheses. ^*^p<0.05; ^**^p<0.01.

Likewise, critical access hospitals had higher odds of having implemented most of the initiatives compared with hospitals without critical access hospital designation. Hospital size and community-level characteristics, on the other hand, were largely unrelated to the extent to which hospitals had implemented any of the health equity initiatives included in the AHA Annual Survey.

The zero-truncated Poisson regression analysis showed that larger hospitals tended to have more comprehensive health equity strategic plans than smaller hospitals (Table 3) and nonprofit hospitals tended to have more comprehensive health equity strategic plans than for-profit and government hospitals.

**Table 3.** Organizational and Community-Level Characteristics Associated with the Comprehensiveness of Hospitals' Health Equity Strategic Plans

	Coefficient (robust standard error)
Organizational characteristics	
Bed size	0.000084^*^ (0.000038)
Ownership	
Nonprofit	Reference group
For-profit	−0.17^**^ (0.045)
Government	−0.14^**^ (0.039)
System member	0.27^**^ (0.033)
Critical access hospital	0.098^**^ (0.033)
Community-level characteristics	
Median household income	0.0000012^*^ (0.00000057)
Uninsured rate for those younger than 65 years	−0.00040 (0.0024)
% Black residents	−0.00062 (0.00094)
% Hispanic residents	0.0020^**^ (0.00076)
Urban–rural location	
Rural location	Reference group
Urban location	0.061^*^ (0.029)

Note: Table shows coefficients obtained through zero-truncated Poisson regression analysis with robust standard errors in parentheses. ^*^p<0.05; ^**^p<0.01.

Similarly, system-affiliated hospitals had more comprehensive health equity strategic plans than hospitals not affiliated with a health system, as did critical access hospitals. Community-level characteristics associated with more comprehensive health equity strategic plans included county-level household income and urban location, as well as the proportion of county residents who are Hispanic.

## Discussion

Gaining insight into the present state of health equity initiatives in hospitals represents a crucial step toward enhancing health and promoting equity in communities throughout the United States. The 2021 AHA Annual Survey yielded comprehensive data from nearly 2000 hospitals, shedding light on hospitals' efforts in this area. Hospitals revealed their most common initiatives, which included “community engagement,” “culturally appropriate patient care,” and “equitable and inclusive organizational policies.”

Slightly less prevalent initiatives included “diverse representation in hospital and health care system leadership,” “collection and use of segmented data to drive action,” and “diverse representation in hospital and health care system governance.” The least frequently mentioned initiative was “systematic and shared accountability for health equity.” In general, over half of the respondents were involved in five or more health equity activities, indicating that a growing number of hospitals are acknowledging the significance of addressing health equity in their strategic plans.

Organizational characteristics play an important role in the extent to which hospitals adopt comprehensive health equity strategic plans. Regression analysis showed that larger hospitals, nonprofit hospitals, and hospitals affiliated with health systems were more likely to include health equity initiatives in their strategic plans.

Larger hospitals and hospitals affiliated with systems often have more dedicated staff and more resources to dedicate to health equity initiatives than smaller hospitals and independent hospitals. Health systems often have centralized offices to provide guidance and resources and support member hospitals in their efforts to develop and implement health equity initiatives.

Hospital ownership mattered, too. In return for tax exemption, nonprofit hospitals are required to conduct periodic community health needs assessments, develop implementation strategies, and report their spending on various categories of community benefit as part of their annual tax return (IRS Form 990 Schedule H). The continued focus on community health of nonprofit hospitals likely contributed to the greater commitment of these hospitals to developing and implementing health equity initiatives.

Government hospitals, on the other hand, had less comprehensive health equity strategic plans when compared with nonprofit hospitals. While government hospitals often serve communities with substantial health needs, resource constraints may prevent them from engaging more fully in health equity-related work.

The extent to which organizations embraced comprehensive health equity strategic plans was influenced by the community in which a hospital was situated. Hospitals located in urban areas and those within counties with higher median household incomes exhibited a greater likelihood of adopting comprehensive health equity plans. Similarly, hospitals situated in communities with a higher percentage of Hispanic residents were more inclined to adopt such plans.

The finding that the community in which a hospital is situated influences the adoption of comprehensive health equity strategic plans sheds light on the complex interplay between health care institutions and their local environments. Urban hospitals, for instance, tend to serve more diverse populations and often face unique health challenges. These facilities are likely to have a stronger impetus to address health equity issues due to the presence of marginalized communities, ethnic diversity, and higher population density.

Hospitals located in counties with higher median household incomes may have greater access to financial resources, which can facilitate the development and implementation of comprehensive health equity plans. These hospitals might also perceive a stronger demand from their communities to address health disparities, given the resources available.

Finally, hospitals in communities with higher proportions of Hispanic residents might be more attuned to the unique health care needs and cultural preferences of this demographic group. This awareness could drive them to adopt health equity plans that are specifically tailored to address disparities affecting Hispanic populations.

### Limitations

This study has several limitations that readers should keep in mind when interpreting the findings. First, data on hospitals' health equity initiatives were available for only 1 year (2021), which was the first year for which the AHA Annual Survey collected this type of information. Future analysis of multiple years of data is needed to gain a better understanding of the types of health equity initiatives that hospitals include in their strategic plans.

Second, the survey questions only asked respondents to indicate whether or not their organization included a given health equity initiative in its strategic plan. Binary response choices do not allow respondents to share more detailed information on the specific type of action a hospital may take as they implement health equity initiatives. A more in-depth analysis of hospitals' efforts to increase health equity would require additional data collection using interviews, focus groups, or surveys.

Third, with the exception of “community engagement,” the AHA survey focused on health equity initiatives that target structures and processes within the four walls of their organization. The survey questions do not address upstream factors of health, nor do they align with a comprehensive framework for health equity action. A more comprehensive picture of hospitals' activities in this area requires inclusion of activities beyond the institution aimed at addressing health equity. In addition to community outreach and engagement, hospitals can play a vital role in advocacy, using their influence to promote systemic transformations aimed at improving health equity.

Finally, fewer than half of all general medical and surgical hospitals that completed the 2021 AHA Annual Survey provided data on their health equity initiatives. As shown in Table 1, hospitals with missing data differed significantly from hospitals with complete data. As a result, the findings of this study cannot be generalized to all general medical and surgical hospitals.

Given the substantial amount of missing data for smaller hospitals, independent hospitals, and rural hospitals, future work is needed to shed additional light on the types of health equity initiatives that these hospitals engage in and the organizational partners accountable for achieving these goals.

## Conclusions

Enhancing health and promoting health equity in communities nationwide require integration of health equity initiatives into the strategic plans of hospitals, marking a crucial stride in the right direction. However, the mere presence of a strategic plan does not guarantee effective and efficient implementation of these initiatives.

To ensure that health equity efforts effectively address the most pressing community needs and are carried out in collaboration with various community partners, it is imperative to foster partnerships between hospitals and other community entities. Collaborative initiatives such as joint community health needs assessments and implementation planning play a vital role in facilitating this.

Many state and local health departments have been conducting community health assessments and developing community health improvement plans, following the 10 Essential Public Health Services framework and as a requirement for accreditation by the Public Health Accreditation Board. Hospitals can potentially work together with government public health agencies in their communities during this process.

Furthermore, smaller hospitals and those without the advantages of being affiliated with a health system may require additional resources to support their health equity initiatives. To address this disparity, professional associations such as the AHA can assume a pivotal role. They can provide valuable assistance by offering their expertise and guidance to hospitals through educational programs and specialized training geared toward enhancing organizational capacity within these health care institutions.

In addition to these resources, fostering collaboration with community partners becomes crucial for smaller hospitals. This includes building strong relationships with other health care providers, public health agencies, and local community nonprofits. By working together, health care institutions can leverage each other's strengths, share resources, and create a more comprehensive and sustainable approach to tackling health equity issues within their communities.

Finally, further research is required to more fully understand the effects of hospitals' investments in health equity initiatives on health outcomes. While initial evidence suggests that hospitals with well-developed programs targeting the social determinants of health have achieved improved outcomes, it is crucial to place greater emphasis on evaluating the return on investment for these programs as they become more prevalent throughout the United States.^10^

## Abbreviations Used

AHA: American Hospital Association
AHRF: Area Health Resources Files
CMS: Centers for Medicare and Medicaid Services
HRSA: Health Resources and Services Administration
SD: standard deviation
